# Adaptation and Validation of the Scale of Types of Users in Gamification with the Spanish Adolescent Population

**DOI:** 10.3390/ijerph17114157

**Published:** 2020-06-10

**Authors:** Ana Manzano-León, Pablo Camacho-Lazarraga, Miguel A. Guerrero-Puerta, Laura Guerrero-Puerta, Antonio Alias, Rubén Trigueros, José M. Aguilar-Parra

**Affiliations:** 1 Hum-878 Research Team, Health Research Centre, Department of Psychology, University of Almería, 04120 Almería, Spain; aml570@ual.es; 2 Centro Universitario San Isidoro, Seville, 41092 Spain; pcamacho@centrosanisidoro.es; 3 Department of Education, University of Seville, 41004 Seville, Spain; migupu97@gmail.com (M.A.G.-P.); laura.guerrero.puerta@gmail.com (L.G.-P.); 4 Department of Education, University of Almería, 04120 Almería, Spain; aag344@ual.es; 5 Department of Language and Education, University of Antonio de Nebrija, 28015 Madrid, Spain

**Keywords:** psychometric properties, type of players, game-design, adolescence

## Abstract

The video game has been one of the phenomena with the greatest impact on the entire social fabric, and especially among young people. Therefore, it is essential to understand the interaction between the players and the video game itself. However, few instruments have been designed to assess the types of players in the adolescent population. Therefore, the purpose of this study was to validate the Gamification User Types Hexad Scale for the Spanish adolescent population. The sample of participants consisted of 1345 adolescents between the ages of 13 and 18. To evaluate the psychometric properties of the scale, a confirmatory factor analysis and a multi-group analysis of invariance by sex were performed. The results provide evidence of a valid and reliable six-factor instrument to measure the types of players in the Spanish adolescent population, regardless of their sex.

## 1. Introduction

The video game has been one of the phenomena with the greatest impact on social and cultural spheres from 1970 to the present day [1]. It is considered the main driver of global entertainment today, representing an industry that has generated $134.9 billion in 2018 and is expected to grow at an average annual rate of 9.3% by 2021 [2]. Video games have become the preferred game for children, adolescents, and adults, influencing their leisure, socialization, and culture [3]. For this reason, several studies have suggested the importance of designing games that meet the needs of the greatest number of users possible [4], as well as the social and psychological benefits of using video games [5].

Therefore, it is especially important to understand the interaction between players and the game design itself. Currently, research is being carried out on custom video game designs and gamification according to the users to be worked with. In relation to custom designs, game design consists of creating playful activities that generate a perceived challenge that is sufficient for the players to enjoy and get involved [6]. When implementing playful activities, it must be taken into account that not all users will be motivated only to win, but that there will be other interests within the game experiences such as cooperating, collecting items, exploring, etc. “The player’s experiences with a game can be much more extensive than those of a user with a traditional interactive system, which requires reflection on a series of properties that identify and measure these experiences” [7]. Recognizing the different interests and motivations of players helps to develop an engaging environment. In this way, it is possible to incorporate elements, mechanics, and game dynamics that are more conducive to the participation of each user in the activity.

This study aims to adapt and validate a scale to assess the profiles of players in Spanish adolescent population and context, allowing researchers to use it to individualize and improve their interventions in game experience design.

## 2. Adolescents’ Motivations for Video Games

Numerous studies indicate that video games are part of the daily routine of children and adolescents [8]. Their main motivations for playing video games are separated into social motivations (focus for hanging out, joy of competition, teaching each other, making friends, and opportunities to lead), emotional motivations (flow and emotional regulation) and intellectual and expressive motivations (experimentation, challenge, creativity, and discovery) [9].

Video games can report negative consequences at these ages such as addiction and aggressiveness if exposed to inappropriate content or they play games for an excessive amount of time [10]. However, in recent years the benefits of gaming and gamification in teens are being explored. In this sense, the design of a playful experience must encourage behaviors that facilitate the best results in the system and involve the greatest number of users possible [11], and knowing the types of dominant players allows the redesign of the proposed activities so that they have a greater impact on immersion, mastery and flow [12]. For the adolescent community, playful experiences have not gone unnoticed. This methodological strategy has been used in different settings such as health and well-being, for example to involve adolescents in adopting healthier lifestyle behaviors [13]; socio-cultural to raise awareness of the proper use of social networks [14] or therapeutic as gamma applications of mental health [15]. Finally, in the educational field, the use of gamification [16], educational games [17], and playful strategies such as modeling through applications with a game interface such as Classdojo or Classcraft [18] are being studied as learning tools that provide an attractive and motivating approach due to their ability to teach and reinforce curricular content and skills.

## 3. Type of Players: How to Understand Human Behavior in Video Games?

There are different classifications of players, the most commonly used being the one proposed by Bartle [19]. According to their classification, we can differentiate between four types of players (Achievers, Explorers, Socializers, and Killers), according to their actions, motivation, and interests within the game: Achievers seek to act with the game, as long as it is shared with other people and has recognition strategies (medals, badges, etc.) or can unlock achievements. Explorers seek to interact with the world provided in the game and explore it. They have a great eagerness to discover and are interested in knowing the whole game. Socializers are interested in interacting with other players and developing a network of friends and contacts, they show greater engagement in cooperative games where direct interaction with other users is allowed. Killers are interested in acting and competing with other people with the aim of demonstrating their superiority, they only show interest in competitive games and elements such as leaderboards or rankings.

Based on the theory of Bartle [19], other player classifications were developed such as the BrainHex model [20]. BrainHex presents seven archetypes of players inspired by neurobiological research: Seeker: is motivated by a mechanism of interest, which is related to the area of the brain that processes sensory information and memory association. Survivor: enjoys the intensity of an experience associated with terror thanks to the neurotransmitter epinephrine, which increases the effects of dopamine, which is activated when rewards are received. Daredevil: is related to the emotion of chasing and taking risks. Related behavior focuses on the pursuit of emotion and is also associated with epinephrine. Mastermind: They enjoy solving puzzles and developing strategies to make the most efficient decisions, for them every time they are faced with a puzzle, the brain’s decision center and the close relationship between it and the pleasure center ensures that it is an inherently rewarding activity. Conqueror: They enjoy winning against enemies or challenging players. When faced with difficult situations, their body produces epinephrine and norepinephrine, producing excitement and persistence in the face of challenge. Socializer: Their main source of fun is relating to other players. This behavior is connected to the social center and is the main neural source of oxytocin; Achiever: His motivation is long-term goals and achievements. It is based on dopamine.

Marczewski [11] formulated the RAMP model, an acronym for the four basic inducers of intrinsic motivation: Relatedness, Autonomy, Mastery, and Purpose. The RAMP model is of great interest and importance in understanding player behavior and motivation.

RAMP is made up of the following acronyms: Relatedness would represent the desire to be related or connected with others. The fidelity of a community, for example, can be maintained if its members naturally seek interactions. Autonomy is directly related to the feeling of freedom, which is of great importance when it comes to intrinsic motivation. The ability to decide or organize oneself are valued elements in creative activities. Mastery is the process by which a manifest ability is acquired for the development of a specific type of activity. This phenomenon is related to the concept of flow [n], which is obtained with the balance between the sensation of increasing our expertise in direct relation to the level of challenge we face. The importance of this concept in the game lies in the need for there to be challenges for assumable users with a reasonable effort in order to maintain their motivation in the system in which they operate. Purpose is the need to find meaning in the actions we carry out. Users look for a transcendent reason in their activity, even if it does not always have a direct benefit for themselves.

In this model Marczewski separates users into 6 basic types, depending on their predisposition to play: Socializers, whose main motivation is Relatedness, want to interact with others and create social connections; Free Spirits, whose main motivation is Autonomy and self-expression, want to create and explore; Achiever, whose main motivation is Mastery, want to learn new things and challenges to overcome; Philanthropists, whose main motivation is Purpose and Meaning, do not seek rewards within the game, but interact and enrich the lives of other players; Players, whose main motivation is Rewards, play to get all the merits of the game; Disruptors, whose main motivation is Change, in general, want to disrupt their system, either directly or through other users to force a positive or negative change.

To address the need to evaluate user types based on their interactions with game systems, there are various instruments to assess the motivation of an adult player when playing video games. For example, Gameplay Activity Inventory [21] where its latent factors are Aggression, Management, Exploration, Coordination, and Caretaking. Additionally, The Trojan Player Typology [22], where six types of player motivations are mentioned: socializer, completionist, competitor, escapist, story-driven, and smarty-pants. Tondello, Mora, Marczewski, and Nacke [23] designed the Gamification User Types Hexad Scale, which establishes a user preference profile for interaction with the game. The scale consists of 24 items distributed equally among six factors: philanthropic, socializing, free spirit, winner, disruptor, and player. These items were answered through a Likert scale ranging from 1 (strongly disagree) to 7 (strongly agree). The authors used a sample of 556 adults, 323 men, 224 women, and 9 people who did not report their sex (M = 30.37, SD = 10.07) to validate the scale. The structural validity of the scale is generally acceptable by reliability analysis and factor analysis. In this sense, in the exploratory factor analysis, the correlation matrices were adequate, with a KMO (Kaiser-Meyer-Olkin test) = 0.746 for the English sample and KMO = 0.844 for the Spanish sample) and the Bartlett sphericity test was significant for both samples (χ^2^ (276) = 1782.1, *p* < 0.001 for the English sample; χ^2^ (276) = 3771.9, *p* < 0.001 for the Spanish sample).

## 4. Objective and Hypothesis

Based on this background, this paper aims to adapt and validate the Gamification User Types Hexad Scale instrument of Tondello, Mora, Marczewski, and Nacke [23] in Spanish teenagers in order to know the player profiles to be able to design playful experiences according to their interests. In research and programs where the focus of study is on gamification strategies, escape rooms, breakouts, or game based learning, this scale will be able to predict the types of players that are users and, consequently, it will be possible to design the game-playing strategy so that it favors the motivation of the users. For this study, this scale has been selected for two reasons, firstly its relationship with the RAMP model and the correct selection of types of players and, secondly, because their study had a Spanish sample, which we consider may be of benefit as it can be validated for Spanish adolescents. To this end, a confirmatory factor analysis will be performed to analyze the factor structure of the questionnaire and a reliability analysis will be carried out. It is expected that the questionnaire will be reliable, valid, and that it will show gender invariance.

## 5. Method

### 5.1. Participants

The participants in study one were 1345 young people (675 males and 670 females) aged 14–18 (M = 15.73, SD = 1.12) from two secondary schools in Almeria (Spain).

### 5.2. Measurements

Hexadecimal Scale of Gamification User Types. The Gamification User Types Hexad Scale version of Tondello, Mora, Marczewski, and Nacke was used [23]. The scale consists of 24 items distributed equally among six factors: philanthropic, socializing, free spirit, winner, disruptor, and player. Study participants were asked to respond according to a Likert scale ranging from 1 (strongly disagree) to 7 (strongly agree).

### 5.3. Procedure

In order to validate the questionnaire in the Spanish context, the reverse translation strategy was used [24]. This process consists of the original questionnaire being translated into Spanish by a group of expert translators and subsequently translated into the original language by another group. The accuracy of the translation was judged by the degree of agreement with the original version. The version obtained was analyzed by three psychologists with extensive research experience [25], in such a way as to ensure that the items obtained were well designed to measure the construct to be measured, without losing the original meaning.

Once the questionnaire was obtained, various secondary schools were contacted and informed of the objective of the investigation and their collaboration was requested. Subjects were required to have the permission of their parents or legal guardian for their voluntary participation. The administration of the questionnaire was carried out under the supervision of an expert pollster who was a member of the research group, who explained and resolved the doubts that arose when completing it. The estimated time to complete the questionnaire was around 15 min. The study was approved by the university’s ethics committee prior to data collection.

### 5.4. Data Analysis

In order to determine the validity and reliability of the questionnaire in the Spanish context, the psychometric properties of the instrument were analyzed. First, a confirmatory factor analysis (CFA) was performed to test the factor structure. Secondly, multi-group analysis was carried out to analyze gender invariance. Descriptive statistical analyses were then performed and the reliability of the instrument was tested through internal consistency analysis (Cronbach’s alpha). The statistical packages SPSS 25.0 (IBM, Armonk, NY, USA) and AMOS 20.0 (IBM, Armonk, NY, USA) were used for the data analyses.

Because the Mardia coefficient was found to be high (196.67) for the CFA, the maximum likelihood estimation method was used along with the bootstrapping procedure. In order to accept or reject the model tested, a set of adjustment indexes was considered: *χ*^2^*/df*, CFI (Comparative Fit Index), IFI (Incremental Fit Index), RMSEA (Root Mean Square Error of Approximation) plus its confidence interval (CI) at 90%, and SRMR (Standardized Root Mean Square Residual). Since *χ*^2^ is very sensitive to sample size [26], *χ*^2^*/df* was used, and values less than 5 [27] were considered acceptable. Incremental indices (IFC and IFI) show a good fit with values equal to or greater than 0.95 [28], while error rates (RMSEA and SRMR) are considered acceptable with values equal to or less than 0.08 [29,30].

## 6. Results

### 6.1. Confirmatory Factor Analysis

**Figure 1:**
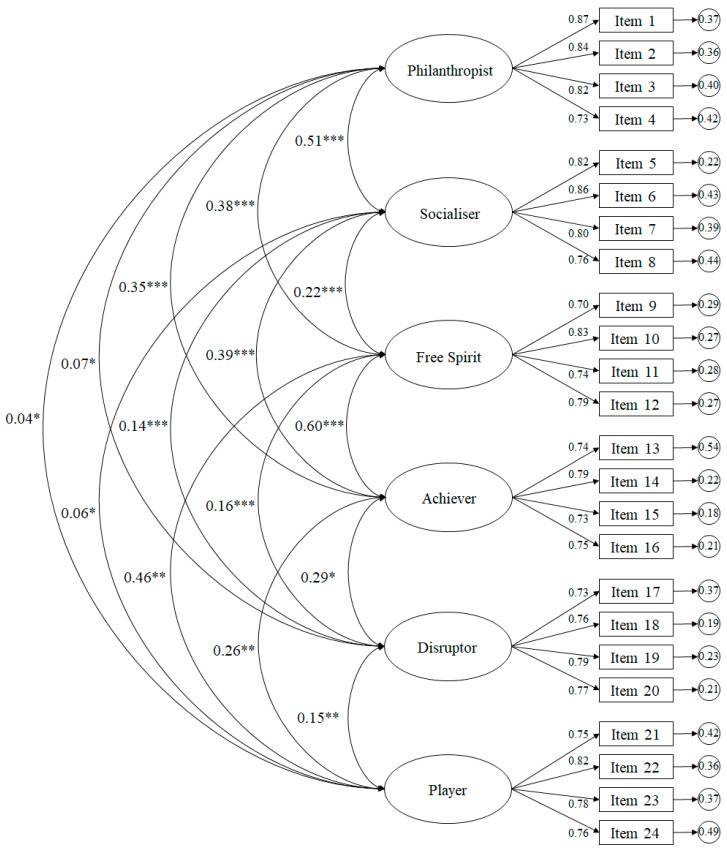
Confirmation factor analysis of the questionnaire. The ellipses represent the factors and the rectangles represent the different items. The residual variances are shown in the small circles. * *p* < 0.05, ** *p* < 0.01, *** *p* < 0.001.

The fit ratings of the model tested (Figure 1) were appropriate: χ^2^ (1237. N = 325) = 643.57, *p* < 0.01; *χ*^2^*/df* = 2.78; IFC = 0.95; IFI = 0.95; RMSEA = 0.071 (CI 90% = 0.064–0.077); SRMR = 0.049. The standardized regression weights were between 0.60 and 0.91, being statistically significant (*p* < 0.001).

### 6.2. Gender Invariance Analysis

To find out whether the factor structure of the scale is invariant to gender, a multi-group analysis was carried out. As shown in Table 1, no significant differences were observed in the statistic χ^2^ between model 1 (unrestricted model) and model 2 (model of invariance in the measurement weights). On the other hand, the results did show significant differences between model 1 and model 3 (structural invariant covariance model) and model 4 (residual invariant measurement model). The minimum criterion to accept that the factor structure of the questionnaire is invariant, the absence of significant differences between model 1 and 2 [31].

### 6.3. Descriptive and Reliability Statistics

Table 2 shows the various statistical analyses carried out, half standard deviation and Cronbach’s alpha, with positive correlations between the study factors. In addition, an internal consistency analysis (Table 2) was performed, revealing a Cronbach’s alpha value greater than 0.70 for each of the users of the gamification.

## 7. Discussion

Gaming systems are effective when they help users achieve their goals, which often involve the acquisition of knowledge, support their changes in attitude or behavior, or focus their interest on specific issues [32]. The aim of this study is to adapt and validate the Gamification User Types Hexad Scale to the Spanish adolescent population. The validation of this questionnaire may be important, since it would help to understand and deepen the theory of game-design and design of recreational experiences for adolescent.

The results of this study have shown that it is an instrument that shows evidence of validity and reliability to measure different types of players. In this sense, the confirmatory factor analysis showed adequate psychometric properties and that the factor structure of the questionnaire is composed of six factors. These results are similar to those of the original Tondello, Mora, Marczewski, and Nacke scale [23] where an exploratory factor analysis showed that the questionnaire was composed of six factors. Furthermore, the invariance analysis showed that the questionnaire is understood in the same way by boys and girls. However, these results cannot be compared with those of the original scale since the authors did not contemplate such an analysis. Thus, the present study shows evidence that both boys and girls understand each of the items in the same way, so that future studies can be conducted to compare both populations.

Descriptive statistical analyses and reliability analyses show a positive correlation between factors in line with the results achieved in confirmatory factor analysis. The reliability analysis each of the factors reaches a Cronbach’s alpha score above 0.70 which shows that the distribution of the items is adequate [24]. As in the original study, this phenomenon can be explained through the different taxonomies of players. Among them, Amy Jo Kim’s model of players, where motivational patterns in games are classified, associating them with social loyalty verbs: explore, create, compete, and collaborate [33], seeking to define users based on what they enjoy doing. This theory suggests that there is no single player profile, and shows the overlap between types of players, since different profiles may show a main interest in the same loyalty verb, differentiating themselves by other secondary verbs in order to know the particularities and desires within the game system and playful experience of each user.

Despite the results achieved, there are some limitations that should be mentioned. Firstly, the sample has been non-probabilistic and belongs to the same city, and, consequently, the results cannot be generalized to other groups of people. Secondly, factor analysis has shown evidence that the instrument can be used regardless of gender, however, future work should determine whether it can also be used to establish differences in the type of player according to other variables (e.g., age, educational status, socio-economic level). In addition, it may be useful to validate a short version of the scale following the recommendations of Ziegler, Kemper, and Kruyen [34] to determine the type of player in game situations.

The objective of this study has been to validate the Gamification User Types Hexad Scale for adolescent population. The next steps will be: On the one hand, to perform an ethnographic study to find out the most common types of players in the Spanish adolescent population. On the other hand, to design and evaluate an educational gamification program that considers student’s type of players, in order to investigate whether the design of the gamification can influence school motivation.

## 8. Conclusions

In short, the results of this study support the Gamification User Types Hexad Scale as a valid and reliable instrument to measure the types of players in the Spanish adolescent population. Tondello, Mora, Marczewski, and Nacke [23] designed the Gamification User Types Hexad Scale, with the aim of establishing a profile of user preferences to interact with the game. This study has shown evidence that the instrument (Table A1) can be used to identify the types of players in an adolescent population when designing and implementing gaming experiences. This instrument will allow the analysis of the different motivations (philanthropic, socializing, free spirit, winner, disruptor, and player) of the adolescent users in the interaction with the game, showing empirical evidence that can later be related to the engagement and motivation of young people in playful activities.

## Figures and Tables

**Table 1:** Gender Invariance Analysis.

Models	*χ* ^2^	*df*	*χ* ^2^ */df*	∆*χ*^2^	∆*df*	CFI	IFI	SRMR	RMSEA (IC 90%)
Model 1	958.48	474	2.02	-	-	0.95	0.95	0.041	0.055 (0.049–0.060)
Model 2	985.06	492	2.00	26.58	18	0.95	0.95	0.042	0.054 (0.049–0.060)
Model 3	1031.65	513	2.01	73.46 **	39	0.95	0.95	0.042	0.054 (0.050–0.060)
Model 4	1095.23	537	2.04	136.74 ***	63	0.94	0.94	0.042	0.055 (0.050–0.060)

Note: ** *p* < 0.01; *** *p* < 0.001.

**Table 2:** Descriptive statistics, reliability analysis, and bivariate correlations.

Factors	*M*	*SD*	Range	α	1	2	3	4	5	6
1. Philanthropic	5.99	1.04	1–7	0.82	-	0.49 ***	0.32 ***	0.33 ***	0.04	0.14 ***
2. Socializer	5.49	1.08	1–7	0.81			0.17 ***	0.32 ***	0.07	0.11 ***
3. Free Spirit	5.70	0.82	1–7	0.85				0.44 ***	0.34 ***	0.14 ***
4. Winner	5.53	0.95	1–7	0.83					0.21 ***	0.23 ***
5. Disruptor	3.76	1.25	1–7	0.84						0.14 ***
6. Player	4.45	1.21	1–7	0.77						

Note: *** *p* < 0.001.

## Appendix A

**Table A1:** This questionnaire was validated in Spanish.

1	Me hace feliz poder ayudar a los demás *It makes me happy if I am able to help others*
2	Me gusta ayudar a otros a orientarse en nuevas situaciones *I like helping other to orient themselves in new situations*
3	Me gusta compartir mis conocimientos *I like sharing my knowledge*
4	El bienestar de los demás es importante para mí *The wellbeing of others is important to me*
5	Interactuar con otras personas es importante para mí *Interacting with others is important to me*
6	Me gusta ser parte de un equipo *I like being part of a team*
7	Es importante para mí sentir que soy parte de una comunidad *It is important to me to feel like I am part of a community*
8	Me gustan las actividades grupales *I enjoy group activities*
9	Es importante para mí seguir mi propio camino *It is important to me to follow my own path*
10	A menudo dejo que me guíe mi curiosidad *I often let my curiosity guide me*
11	Ser independiente es importante para mí *Being independent is important to me*
12	Me gusta poder expresarme en los juegos *I like being able to express myself in games*
13	Me gusta superar obstáculos *I like overcoming obstacles*
14	Es importante para mí completar siempre todas mis tareas *It is important for me to always complete all my tasks*
15	Es difícil para mí dejar un problema antes de encontrar su solución *It is difficult for me to leave a problem before finding its solution*
16	Me gusta dominar las tareas difíciles *I like mastering difficult tasks*
17	Me gusta provocar *I like to provoke*
18	Me gusta cuestionar el status quo (lo establecido en la sociedad) *I like to question the status quo*
19	Me veo como un rebelled *I see myself as a rebel*
20	No me gusta seguir las reglas *I dislike following rules*
21	Me gustan las competiciones donde se puede ganar un premio *I like competitions where a prize can be won*
22	Las recompensas son una buena manera de motivarme *Rewards are a great way to motivate me*
23	Recuperar lo invertido es importante para mí *Return of investment is important to me*
